# Inhibitory Potency of 8-Methoxypsoralen on Cytochrome P450 2A6 (CYP2A6) Allelic Variants CYP2A6*15, CYP2A6*16, CYP2A6*21 and CYP2A6*22: Differential Susceptibility Due to Different Sequence Locations of the Mutations

**DOI:** 10.1371/journal.pone.0086230

**Published:** 2014-01-27

**Authors:** Kai Hung Tiong, Nafees Ahemad Mohammed Yunus, Beow Chin Yiap, Eng Lai Tan, Rusli Ismail, Chin Eng Ong

**Affiliations:** 1 School of Pharmacy and Health Sciences, International Medical University, Kuala Lumpur, Malaysia; 2 Jeffrey Cheah School of Medicine and Health Sciences, Monash University Malaysia, Bandar Sunway, Selangor, Malaysia; 3 Centre of Excellence for Research in AIDS (CERiA), Universiti Malaya, Kuala Lumpur, Malaysia; 4 Discipline of Pharmacy, Monash University Malaysia, Bandar Sunway, Selangor, Malaysia; Universidade Federal do Rio de Janeiro, Brazil

## Abstract

Human cytochrome P450 2A6 (CYP2A6) is a highly polymorphic isoform of CYP2A subfamily. Our previous kinetic study on four CYP2A6 allelic variants (CYP2A6*15, CYP2A6*16, CYP2A6*21 and CYP2A6*22) have unveiled the functional significance of sequence mutations in these variants on coumarin 7-hydroxylation activity. In the present study, we further explored the ability of a typical CYP2A6 inhibitor, 8-methoxypsoralen (8-MOP), in inhibition of these alleles and we hypothesized that translational mutations in these variants are likely to give impact on 8-MOP inhibitory potency. The CYP2A6 variant and the wild type proteins were subjected to 8-MOP inhibition to yield IC_50_ values. In general, a similar trend of change in the IC_50_ and K_m_ values was noted among the four mutants towards coumarin oxidation. With the exception of CYP2A6*16, differences in IC_50_ values were highly significant which implied compromised interaction of the mutants with 8-MOP. Molecular models of CYP2A6 were subsequently constructed and ligand-docking experiments were performed to rationalize experimental data. Our docking study has shown that mutations have induced enlargement of the active site volume in all mutants with the exception of CYP2A6*16. Furthermore, loss of hydrogen bond between 8-MOP and active site residue Asn297 was evidenced in all mutants. Our data indicate that the structural changes elicited by the sequence mutations could affect 8-MOP binding to yield differential enzymatic activities in the mutant CYP2A6 proteins.

## Introduction

Cytochrome P450 2A6 (CYP2A6) is the best characterized isoform in the CYP2A subfamily [1], [2]. This isoform constitutes up to 15% of total human microsomal CYP proteins, and is known to predominantly participate in the Phase I metabolism of important therapeutic drugs (e.g. valproic acid, pilocarpine, tegafur, fadrozole, ifosfamide, cyclophosphamide, nicotine, tamoxifen, promazine, propofol, and cisapride) and xenobiotics including nicotine and tobacco-specific nitrosamines [1], [3]. Over years, marked variability has been observed in the CYP2A6 expression and activity attributed to the high degree of polymorphism in the genetic code of the isoform [4]–[8]. Genetic polymorphism of CYP2A6 is an area of intense research, with current reported alleles up to 38 variants (as referred to http://www.cypalleles.ki.se/cyp2a6.htm, access date: 6 June 2013). Elucidation of all alleles and global genotyping for CYP2A6 is vital in the sense that the isoform plays a distinctive role in the metabolism of various substrates, especially pharmacologically and toxicologically relevant compounds. In tandem with nicotine and other tobacco-specific carcinogens being established as high-affinity substrates for CYP2A6, much attention has been focused on the toxicological and clinical significance of this isoform in human.

Genetic alterations involving amino acid mutation of CYP genes have a substantial role on the kinetics function of CYP superfamily. Recent findings from our laboratory have unravelled the functional consequences of genetic polymorphisms in several allelic variants of CYP2A6, CYP2A6*15, CYP2A6*16, CYP2A6*21 and CYP2A6*22 [9]. Kinetic analyses of these polymorphic alleles of CYP2A6 indicated that point mutations harboured in these variants have encoded enzymes that were metabolically active toward coumarin oxidation, with the exception of CYP2A6*22, which has markedly reduced but not inactive metabolic activity. Data from this study imply that individual carriers of the homologous CYP2A6*22 allele would be expected to have decreased ability in the biotransformation of coumarin. These data have triggered our interest to further explore on the susceptibility of these variants of CYP2A6 towards the inhibition by 8-methoxypsoralen (8-MOP), a well-characterized inactivator of human CYP2A enzymes [10]. In the present study, we have assessed the inhibitory potency of 8-MOP using the four CYP2A6 allelic variants, expressed in *E. coli*. The mechanism through which the variants exhibit differential inhibition by 8-MOP was rationalized from the molecular perspective based on our molecular docking study of the inactivator on the molecular models of both CYP2A6 wild type and mutants. In addition, current structural information from the CYP2A6 X-ray crystal structure and published homology models were also discussed.

## Materials and Methods

### Materials and Chemicals

Luria-Bertani and Teriffic broth media were purchased from Invitrogen Corporation (Carlsbad, CA, US). Tris base was acquired from Promega (Madison, WI, USA) while acetonitrile and hydrochloride acid were from Fisher Scientific (Pittsburgh, PA, USA). Coumarin, 7-hydroxycoumarin, 8-methoxypsoralen, nicotinamide adenine phosphate (NADP^+^), glucose-6-phosphate dehydrogenase (G6PDH), glucose-6-phosphate (G6P), dimethyl sulfoxide (DMSO) and magnesium chloride were obtained from Sigma-Aldrich (St. Louis, MI, US). The collection of *Escherichia coli (E. coli)* bacterial stocks harbouring the plasmids of the wild type CYP2A6 (pCW-CYP2A6*1), all four individual variants of CYP2A6 (pCW-CYP2A6*15, pCW-CYP2A6*16, pCW-CYP2A6*21 and pCW-CYP2A6*22) and pACYC-oxidoreductase (pACYC-OxR) was previously constructed and prepared in our laboratory [9].

### Establishment of CYP2A6 Monooxygenase Systems in *E*. *coli*

A fully functional P450 monooxygenase system requires collaboration of the CYP of interest in concert with the essential coenzyme, NADPH-CYP oxidoreductase (OxR). Here, we co-expressed the individual CYP2A6 plasmid constructs – pCW-CYP2A6, pCW-CYP2A6*15, pCW-CYP2A6*16, pCW-CYP2A6*21 or pCW-CYP2A6*22, with pACYC-OxR plasmid which allowed for OxR co-expression that was important for CYP2A6 catalytic function. Preparation of membrane fragments expressing holoenzyme protein of respective CYP2A6 construct was in accordance to the established method described previously [11]. Membrane fractions were stored at −80°C in a 1∶1 mixture of pH 7.6 TES (100 mM Tris/0.5 mM EDTA/500 mM sucrose buffer) and ice-cold distilled water before use.

### CYP2A6 Inactivation Assay

Assessment of coumarin 7-hydroxylation is a signature marker for CYP2A6 metabolic assay. To evaluate the inhibitory potency of 8-MOP on the kinetic activity of CYP2A6 variants, we adopted a fluorescence-based coumarin 7-hydroxylase assay upon minor modifications of an established protocol [12], [13]. Generally, enzyme reactions were prewarmed for a few (5–10) minutes at 37°C in the presence of 50 μg of individual CYP2A6 variant proteins (final protein concentration of 0.25 mg/ml) and various concentration of 8-methoxypsoralen ranging from 0–5 µM, together with coumarin at the final concentration of 10 µM (the K_m_ value predetermined in the previous kinetic study). NADPH generating system (1 mM NADP, 10 mM G6P, 2IU G6PDH and 5 mM MgCl_2_) in 0.1 M phosphate buffer, pH 7.4 was added subsequently to the incubation mixture to initiate the reactions. Organic solvent used to dissolve coumarin and 8-methoxypsoralen was DMSO and acetonitrile respectively with the final content of solvent in the reaction assay retained at 1% (v/v) or less. Enzyme inhibition reactions were allowed in 25 minutes of incubation and later terminated by 500 mM Tris base. The experimental conditions were selected such that under conditions with varying activities of CYP2A6 proteins, no more than 20% of the substrate was converted to 7-hydroxycoumarin. The fluorescent metabolite was detected by Tecan Infinite™ 200 series microplate reader (Männerdorf, Switzerland) at excitation wavelength 365 nm and emission wavelength 450 nm and further estimated based on the standard curve of 7-hydroxycoumarin which was constructed in the range of 15.63–2000 pmol/well previously (data not shown). Current assay also included control for fluorescent interference which consisted of inhibitor (at a concentration equal to the inhibition incubations) and bacterial control membranes. Fluorescence interference from the control incubation was generally negligible (<20%). In experiments involving pre-incubation, the incubations were carried out as described above except that after a few minutes of prewarming, CYP2A6 was incubated with 8-MOP and NADPH generating system for 10 minutes without coumarin. Coumarin was only added after 10 minutes and incubations proceeded for a further 25 minutes. For corresponding incubations without pre-incubation, all reaction components (CYP2A6, 8-MOP, coumarin and NADPH generating system) were added from the beginning and incubated for a total time of 35 minutes. All reactions were incubated in triplicate with replications in separate experiments. Due to photosensitivity of 8-MOP, the assay was conducted using 96-well plates with black polystyrene wells (black bottom) that came with lid. Furthermore, all the 8-MOP stocks were prepared and stored in amber glass tubes or tubes sealed or covered with proper covering (such as filter paper) to protect it from light.

### Kinetic Analysis

Enzyme kinetic data were analyzed by nonlinear least squares regression analysis software EZ-Fit^TM^ (Perrella Scientific, USA) of which the kinetic parameters, Michaelis-Menten constant (K_m_) was determined over the substrate range studied. The IC_50_ values (concentration of inhibitors required to cause 50% reduction in enzyme activity) for the 8-MOP inhibition studies were interpolated mathematically by SigmaPlot® software (version 2004, Systat Software Inc, USA). All experimental data were expressed as means and standard deviations. Comparison of the means was analysed using Student's *t*-test and one way ANOVA with SPSS (version 11.5, SPSS Inc, Illinois, USA).

### Molecular Docking and Virtual Mutation of CYP2A6

All computational works were performed on Discovery Studio 3.5 (Accelrys Software Inc., San Diego, CA, USA). The X-ray crystal structure of CYP2A6 was retrieved from the Protein Data Bank (PDB ID: 1Z11) [14]. The same binding site was used for all docking works involving 8-MOP. 8-MOP was constructed using ChemBIoDraw Ultra 2012. The file was opened in DS and energy minimization was carried out by CHARMm force field [15]. The optimization protocol was applied in preparation of the protein and docking procedures [16]. The CDOCKER program was subsequently used for the docking. The CDOCKER is CHARMm-based docking algorithm that uses the CHARMm family of force fields and algorithm adopts a strategy involving generation of several initial ligand orientations in the active site of target protein followed by molecular dynamics (MD)-based simulated annealing, and final refinement by energy minimization [17]–[19]. 8-MOP was docked into the protein active sites where the best conformations were obtained by CDOCKER. The top 10 docking poses were ranked according to their dock score function based on the total docking energy including the intra-molecular energy for ligands and the ligand-protein interactions. The pose (conformation) having the highest dock score has the most favourable interaction. This procedure was performed first on the wild type CYP2A6 and subsequently repeated for the mutants.

For the virtual mutation of CYP2A6, the Build Mutant Protocol was used for substitution of amino acid residues. Four mutants were generated (CYP2A6*15, CYP2A6*16, CYP2A6*21 and CYP2A6*22) using 1Z11 as the template by substituting the residues in the protein sequence. Energy minimization for optimization of residue geometry was applied to the mutant proteins using the algorithm of smart minimization until the gradient tolerance (RMS Gradient ∼0.1 kcal/mol/Å) was satisfied. 8-MOP was docked in the same binding site as in the case for the wild type.

## Results and Discussion

Functional characterization of polymorphic CYP variants is imperative in defining the magnitude of genetic alterations in the enzymatic activity. To date, CYP2A6 is known as one of the highly polymorphic CYP enzymes in human. Our group previously constructed several mutant alleles of CYP2A6, namely CYP2A6*15 (carrying K194E substitution), CYP2A6*16 (R203S), CYP2A6*21 (K476R) and CYP2A6*22 (D158E and L160I), and we demonstrated that point mutations in CYP2A6 primary sequence have minimum impact on the catalytic functions except for CYP2A6*22, of which the metabolic activity was evidently reduced in relative to the wild type CYP2A6*1 [9]. Despite low frequency of this variant reported among the Caucasian population [20], these findings have at least indicated that individual carriers of the homologous CYP2A6*22 allele is predicted to have diminished coumarin hydroxylase activity. Even though our study [9] showed that no significant difference in intrinsic clearance for CYP2A6*15, CYP2A6*16 and CYP2A6*21 in coumarin 7-hydroxylation when compared to the wild type, there were however apparent differences in the K_m_ values (Table 1) of these variants. The variable K_m_ values observed for the variants indicate that there are subtle yet significant changes in the active site induced by the mutations present in their primary sequences. To gain more insights into the ligand binding and mechanism of structural and functional changes as a result of the mutations, we further explored the inhibitory effect of the typical CYP2A6 inhibitor, 8-MOP on the variants, and compared the inhibitory effect with the binding affinity for coumarin. The differential ligand binding affinity was further rationalized by virtual mutation of CYP2A6 and docking of 8-MOP into the various CYP2A6 molecular models we have generated.

**Table 1:** The kinetic and inhibition profile of 8-methoxypsoralen on coumarin 7-hydroxylase activity of the wild type CYP2A6, CYP2A6*15, CYP2A6*16, CYP2A6*21 and CYP2A6*22.

*CYP Protein*	*K_m_ (µM)*	*IC_50_ (µM)*	*Fold*
CYP2A6*1 (wild-type)	10.61±1.58	0.19±0.05	1
CYP2A6*15	22.13±1.29**	2.39±0.0**	12.58
CYP2A6*16	10.53±1.45	0.43±0.1	2.26
CYP2A6*21	16.39±0.41**	1.56±0.21**	8.21
CYP2A6*22	31.16±2.22**	1.05±0.21**	5.53

All values are shown as the means ± SD of three independent determinations. The K_m_ values were determined in our previous study [9] and listed here for comparison purpose. The IC_50_ values were determined in the present studies. The last column represents the fold of change in IC_50_ relative to the wild type. Degree of statistical differences between the wild-type and the mutants as determined by one-way analysis of variance: the mean difference is significant at 0.05 level (* p<0.05) and highly significant at 0.01 level (** p<0.01).

**Figure 1:**
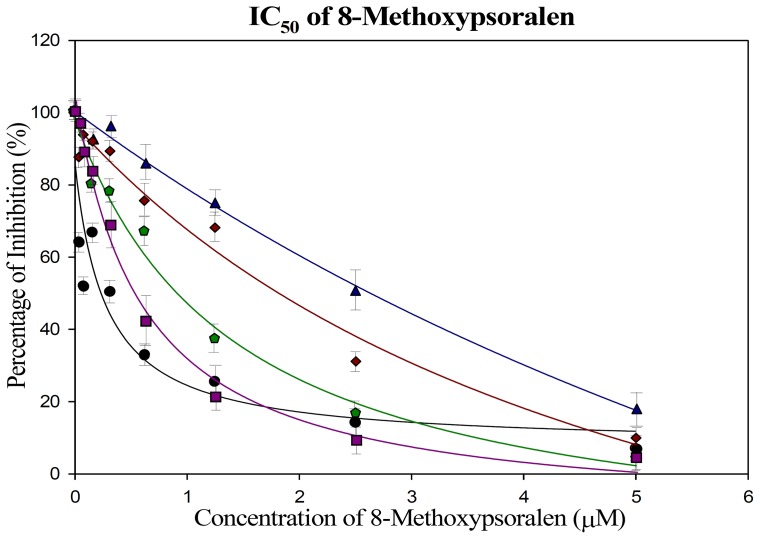
Representative IC_50_ plots demonstrating the inhibitory effect of 8-methoxypsoralen at concentration of 0–5 µM on CYP2A6*1 (wild type) (•), CYP2A6*15 (▴), CYP2A6*16 (▪), CYP2A6*21 (♦) and CYP2A6*22 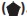. Points are experimentally determined values while solid lines are the computer-generated curves of best fit (by SigmaPlot® programme). Data are presented as mean values from triplicate measurements.

Bacterial membrane fractions expressing human CYP2A6 wild type and variant proteins were exposed to several concentration of 8-MOP ranging from 0–5 µM, according to the protocol described under ‘Materials and Methods’. The inhibitory effect of 8-MOP in coumarin 7-hydroxylation of wild-type and variant CYP2A6*15, CYP2A6*16, CYP2A6*21 and CYP2A6*22 are illustrated in Fig. 1 (A to E) of which IC_50_ values were determined from the curve plots. The IC_50_ value of 8-MOP in coumarin metabolism of wild-type and variants were estimated to be 0.19±0.05, 2.39±0.03, 0.43±0.10, 1.56±0.21 and 1.05±0.21 µM respectively (n = 3). Each value was resolved from the means of three independent experiments. One-way ANOVA test was further performed on the data collected from each CYP2A6 variants. Generally the IC_50_ values of 8-MOP in all mutants was much higher as compared to the CYP2A6*1, with concentration differences up to 12.6-fold noted. Multiple comparisons of IC_50_ values among the variants with the wild type appeared to be highly significant at *p*<0.01. CYP2A6*15, CYP2A6*21 and CYP2A6*22 exhibited IC_50_ values which were 12.6, 8.2 and 5.5 times higher than the wild type value respectively. However, no significant difference was observed for CYP2A6*16, with only a 2.3-fold difference.

**Figure 2:**
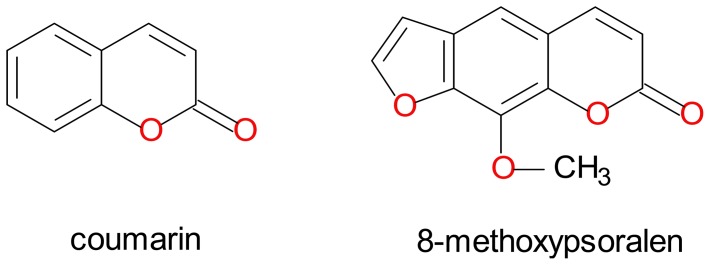
Chemical structures of coumarin and 8-methoxypsoralen.

**Figure 3:**
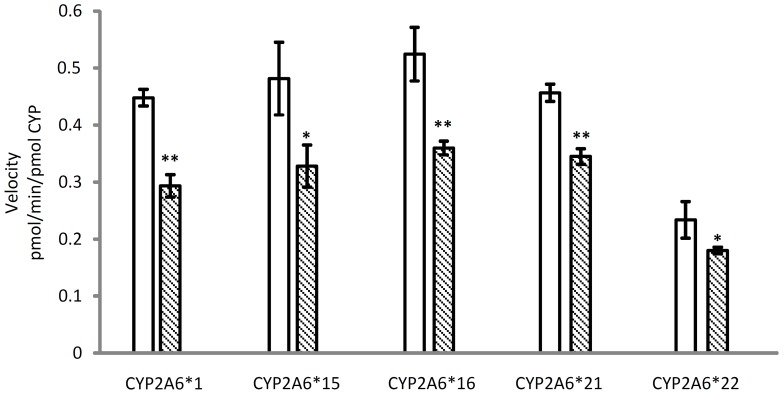
Velocity rates for coumarin 7-hydroxylation in incubations of coumarin (10 µM) without (open bars) or with (hatched bars) pre-incubation with 8-methoxypsoralen at 1.0 µM in CYP2A6 proteins. Data are shown as mean ± SD from triplicate determinations. Degree of statistical differences between incubations without and with pre-incubation as determined by two-tailed Student's *t* test: the mean difference is significant at 0.05 level (* p<0.05) and highly significant at 0.01 level (** p<0.01).

8-MOP is a naturally occurring furanocoumarin in various edible plants [21] with established medicinal value as anti-psoriatic agent [22], [23]. Throughout the years of studies, 8-MOP is notably a potent and relatively selective mechanism-based inhibitor of human hepatic CYP2A6 [10], [24], [25]. Both 8-MOP and coumarin are relatively small planar molecules (Fig. 2) that docked well within the narrow and hydrophobic active site cavity of CYP2A6 [14]. Despite larger in size, 8-MOP was shown to complement the active site cavity without substantially perturbing the configuration for coumarin complex [14]. In general, both molecules adopted similar binding and interaction patterns with side chains of the amino acid residues within the active site cavity. To further explore whether mechanism-based inhibition was also preserved in the four mutants, kinetic analyses of coumarin7-hydroxylation of the proteins were performed with or without a period of pre-incubation with 8-MOP at a fixed concentration of 1.0 µM. As shown in Fig. 3, pre-incubation has resulted in greater degree of inhibition in all four mutants as reflected by the lower velocity rates observed (hatched bars *vs* open bars in Fig. 3). The increased percentages in inhibition were 35, 32, 31, 24 and 23% in the wild type, CYP2A6*15, CYP2A6*16, CYP2A6*21 and CYP2A6*22 respectively. These data have indicated that mutations introduced in the protein sequence did not change the mechanism-based inhibitory nature of 8-MOP toward CYP2A6.

The change in IC_50_ values among the four mutants, in general, show similar trend to the pattern of change in K_m_ values towards coumarin. Statistically significant differences were observed in the IC_50_'s for CYP2A6*15, CYP2A6*21 and CYP2A6*22, all of which also exhibited larger degree of changes in their respective K_m_'s. On the other hand, variant CYP2A6*16 which showed very little change in K_m_ towards coumarin displayed the least degree of increase in IC_50_ value (2.3-fold only). The order of fold difference in CYP2A6*15, CYP2A6*21 and CYP2A6*22 however did not match exactly in both parameters (i.e. fold changes in K_m_ was in the order of CYP2A6*22> CYP2A6*15> CYP2A6*21 whereas the fold changes in IC_50_ was in the order of CYP2A6*15> CYP2A6*21> CYP2A6*22). Exact reason for this is unknown but this may possibly be due to the subtle differences in the molecular structures of coumarin and 8-MOP. 8-MOP is slightly larger in size with two additional functional groups, a methoxy and a furan, in its structure. The additional groups, coupled with the furan oxygen, may have caused slight perturbation of 8-MOP binding with residues within the active site cavity even though both coumarin and 8-MOP have been shown to adopt generally similar binding orientation within CYP2A6.

**Figure 4:**
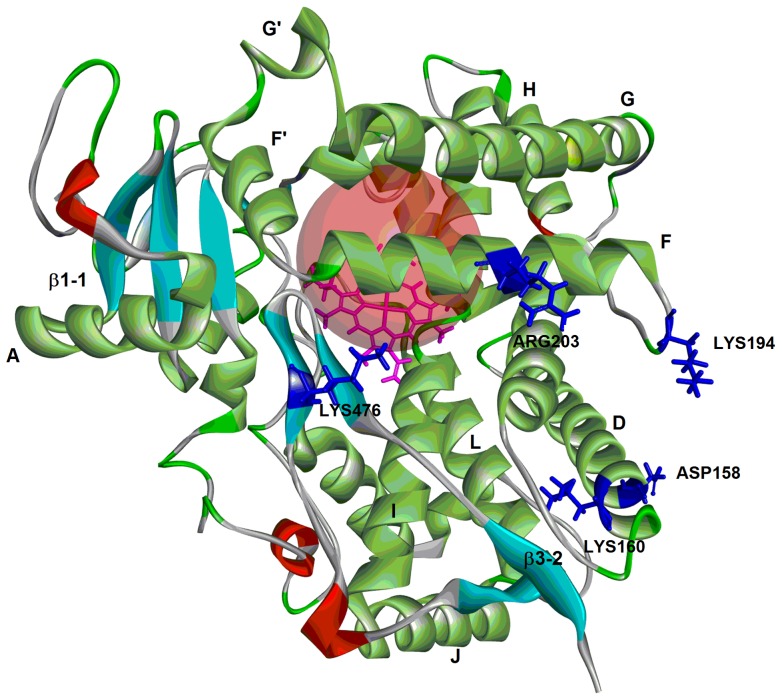
The ribbon diagram showing the overall structure of the wild-type CYP2A6 with locations of mutated residues highlighted. Secondary structures α-helices and β-sheets are also shown and labelled. The heme moiety is indicated in sticks mode (magenta color).

**Figure 5:**
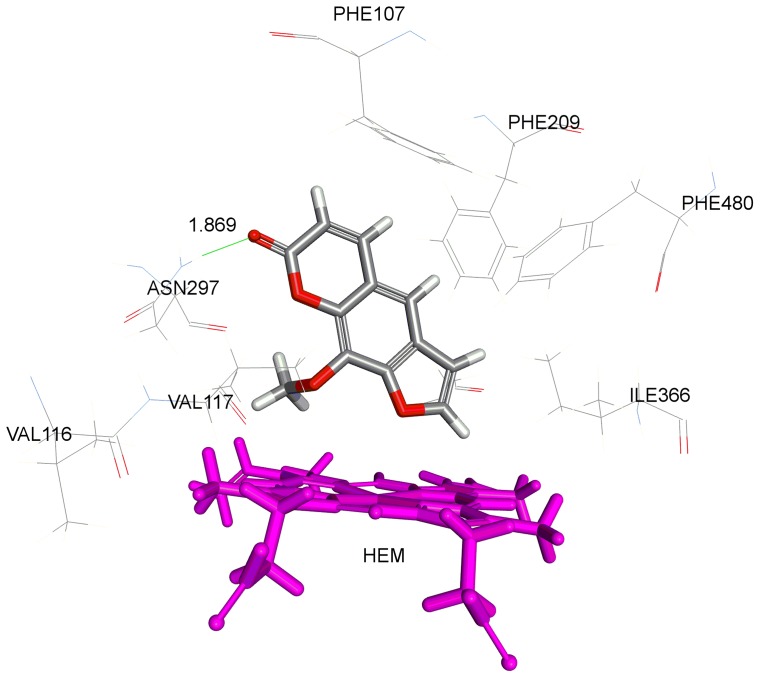
Binding of 8-methoxypsoralen in CYP2A6 wild type active site. Side chains of amino acid residues within 5 Å of the ligand are rendered as stick figures. Red, oxygen atoms; blue, nitrogen atoms; gray, methoxsalen-complex carbon atoms; and magenta, heme group.

**Figure 6:**
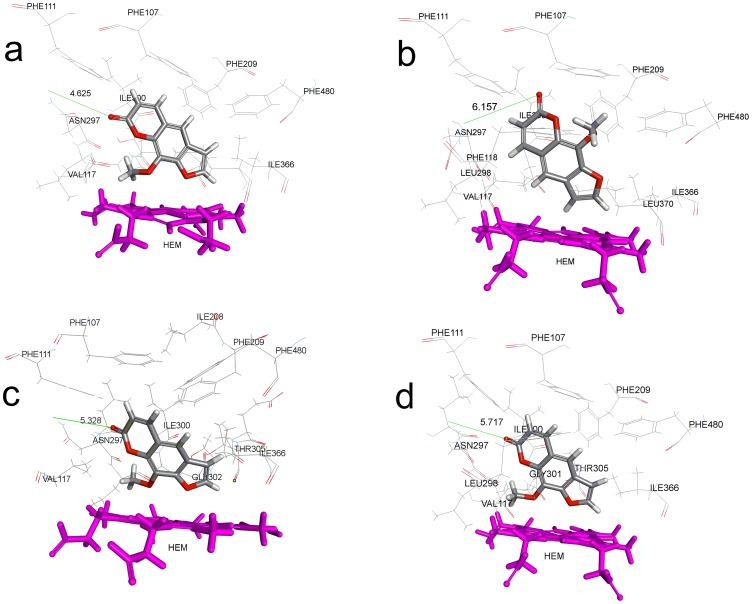
Binding of 8-methoxypsoralen in CYP2A6 mutant active sites. Side chains of amino acid residues within 5 Å of the ligand are rendered as stick figures. Red, oxygen atoms; blue, nitrogen atoms; gray, methoxsalen-complex carbon atoms; and magenta, heme group. a: CYP2A6*15; b: CYP2A6*16; c: CYP2A6*21; and d: CYP2A6*22.

To further explore the molecular factors that determine the binding affinity and binding energy of 8-MOP with CYP2A6 mutants, we conducted detailed molecular docking of the compound to the CYP2A6 active site using the CDOCKER module in DS 3.5. Fig. 4 shows the structural overview of CYP2A6 we constructed based on the published wild type crystal structure (PDB ID: 1Z11) with locations of the six mutations investigated shown. In order to validate docking reliability of our constructed model, 8-MOP was re-docked to the binding site of CYP2A6 and the docked conformation corresponding to the lowest energy was chosen as the most probable binding conformation. As suggested by Yano *et. al.*, CYP2A6 has hydrophobic active site with one hydrogen bond donor, Asn297 that interacts with the carbonyl oxygen of the ligand. This is important key interaction for inhibition of the enzyme [14]. The docked 8-MOP is shown in binding pocket of the enzyme in Fig. 5. The root-mean-square deviation (RMSD) of docked conformation was 0.80 Å, which suggested a high docking reliability of CDOCKER in reproducing the experimentally observed binding mode for CYP2A6 inhibition. The CDOCK interaction energy (CDIE) of −29.17 was determined for the most preferred pose. 8-MOP was subsequently docked in the four CYP2A6 mutant models generated in DS 3.5. The most preferred pose of the ligand for each mutant is shown in Fig. 6 and Table 2 lists the corresponding CDIE values. It is evident from CDIE that wild type protein exhibited the highest binding affinity with the lowest CDIE whereas all mutants showed 1.4 to 3.3 times higher values indicating varying degree of loss in the binding affinity. The rank order of affinity (CYP2A6*1> CYP2A6*16> CYP2A6*22> CYP2A6*15> CYP2A6*21) is in general consistent with the rank order of IC_50_ values in terms of inhibition potency (CYP2A6*1> CYP2A6*16> CYP2A6*22> CYP2A6*21> CYP2A6*15) with the exception of CYP2A6*15 and CYP2A6*21. Nevertheless, these two alleles were ranked last showing that these were the alleles with the most compromised affinity toward 8-MOP. Therefore there is an overall consistency of the *in silico* data to the *in vitro* results. A more remarkable effect observed in all mutants was the loss of the hydrogen bonding between the carbonyl oxygen of 8-MOP with Asn297 (Table 2). As this is the key interaction bond between 8-MOP and CYP2A6, its loss would have a detrimental effect on ligand binding as reflected by both changes in CDIE and experimentally determined IC_50_ values. The distance between 8-MOP carbonyl oxygen and Asn297 in all mutants was further determined and listed in Table 2. As shown in the table, the lengths were 2.5, 3.3, 2.9 and 3.1 times longer than the H bond length of the wild type (bond length of 1.869 Å) for CYP2A6*15, CYP2A6*16, CYP2A6*21 and CYP2A6*22 respectively. This indicates that mutations have resulted in morphological changes within the active site cavity leading to spatial repositioning of active site residues which changed the binding poses of 8-MOP. As illustrated in Fig. 6, 8-MOP was orientated with different poses in different mutants from that of the wild type (Fig. 5) where it adopted slightly different perpendicular angles of its planar structure above the heme plane. The methoxy group was positioned close to the heme iron in CYP2A6*15, CYP2A6*21 and CYP2A6*22 but its orientation in CYP2A6*16 was dramatically changed to the opposite site of the heme plane toward Phe480. In other words, the 8-MOP planar structure has been rotated around 180° in its orientation within the active site cavity. This change in orientation of 8-MOP planar structure has resulted in the longest distance in H bond (6.157 Å) which led to the loss of H bond and reduced binding affinity for this allele. Despite this change in orientation, mechanism-based inhibition was however still observed in CYP2A6*16 (Fig. 3) as were the case of the wild-type and other mutants. This can be rationalized by the fact that the mechanism of inactivation by 8-MOP appeared to be the initial oxidation of the furan ring to generate a furanoepoxide which then reacted with a nucleophilic amino acid at the CYP2A6 active site leading eventually to enzyme inactivation [24], [25]. Therefore in spite of 180° change in 8-MOP orientation, the furan ring was still positioned in close proximity with the heme iron for oxidation in CYP2A6*16, and thus this would still allow furanoperoxide formation and its covalent binding within the CYP2A6 active site.

**Table 2:** Binding interactions and modes of 8-methoxypsoralen to the active sites of the wild type and mutant CYP2A6 proteins.

		*H bond formation*		
*CYP protein*	*CDOCKER Interaction Energy (CDIE)*	*Bond number*	*Residue(s) involved in bonding*	*Distance between 8-MOP carbonyl oxygen and Asn297(Å)*
CYP2A6*1	−29.17	1	Asn297	1.869
CYP2A6*15	−16.00	0	-	4.625
CYP2A6*16	−20.49	0	-	6.157
CYP2A6*21	−8.82	0	-	5.328
CYP2A6*22	−19.60	0	-	5.717

**Figure 7:**
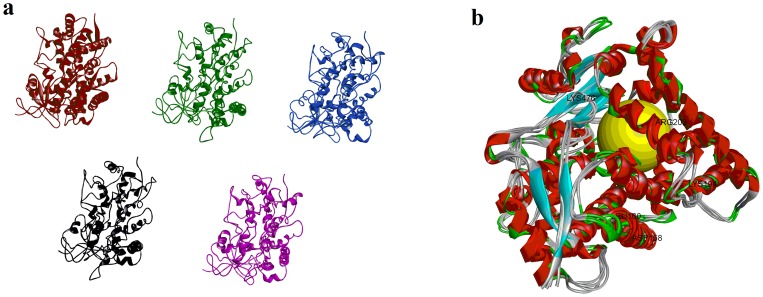
Geometric changes in CYP2A6 wild type and mutant proteins. **a)** Changes in the geometry of the protein tertiary structures. Wild type, red; CYP2A6*15, green; CYP2A6*16, blue; CYP2A6*21, black; CYP2A6*22, magenta; **b)** Superimposition of the wild type and mutant structures. Yellow circle indicates the binding site.

**Figure 8:**
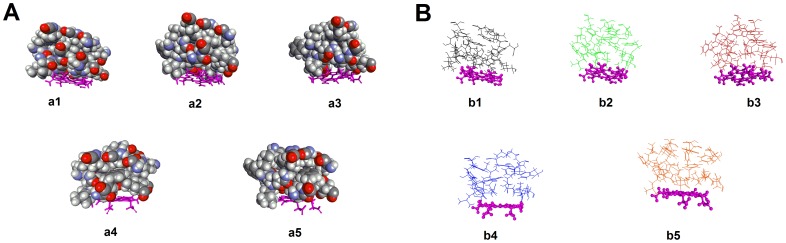
Conformational changes of the CYP2A6 active site cavities. **a)** Space-filling representation of amino acid residues forming the CYP2A6 active sites. a1: wild type; a2: CYP2A6*15; a3: CYP2A6*16; a4: CYP2A6*21; and a5: CYP2A6*22. **b)** The conformations of active site cavities depicted as stick models. b1: wild type; b2: CYP2A6*15; b3: CYP2A6*16; b4: CYP2A6*21; and b5: CYP2A6*22. The heme moiety is presented at the floor of active sites as magenta sticks.

Effect of amino acid substitutions on the active site and overall protein topology was also determined. Remarkable geometric changes in the active site cavity of CYP2A6 were observed. Fig. 7 and Fig. 8 illustrate the overall change in the geometry of the protein tertiary structures and the active sites whereas Table S1 lists the observed topological changes of the active sites for each protein. It is evidenced from Fig. 7 that there was subtle but significant geometric changes due to the mutations introduced into the protein. Spatial repositioning and re-alignment of helical and sheet structures were evidenced when the structures of various mutants were superimposed (Fig. 7b). Fig. 8 illustrates the geometric changes which have taken place within the active site cavity where spatial movement of the active site residues have resulted in different shapes and conformations of the active site cavity. A total of 31 sites were found in the wild type whereas 32, 30, 28 and 28 sites could be searched for CYP2A6*15, CYP2A6*16, CYP2A6*21 and CYP2A6*22 respectively (Table S1). The mutations have caused changes in the active site volume (site 8 in Table S1). CYP2A6*1 showed a volume of 89.56 Å^3^ but the volume was increased in three mutants (105.88 Å^3^ for CYP2A6*15, 130.25 Å^3^ for CYP2A6*21 and 120.75 Å^3^ for CYP2A6*22). This enlarged volume has resulted in 8-MOP adopting different binding orientation as well as losing the H bonding with Asn297 as discussed above. Unlike the other three mutants, the volume for CYP2A6*16 was determined to be 88.25 Å^3^ which was similar to the wild type. Interestingly, this allele has IC_50_ and CDIE values closest to the wild type, indicating that, similar to CYP2A6*1, its active site has assumed a smaller and more compact topology which has allowed a tighter packing interaction and binding with 8-MOP. As discussed above, the R203S mutation in this allele could have induced geometric changes other than expanding cavity volume that have caused the change in 8-MOP orientation within the binding cavity (which assumed the opposite orientation as illustrated in Fig. 6) and the loss of H bond leading to reduced affinity observed. When all these *in silico* data are considered together, the docking data presented are consistent with the *in vitro* data and support the notion that mutations have caused detrimental effect on 8-MOP binding to CYP2A6.

Variant CYP2A6*15 showed the largest IC_50_ and significant larger K_m_ implying the detrimental effect of K194E substitution in both 8-MOP and coumarin binding. This is supported by our docking data that showed enlarged active site volume and loss of H bond. Although this residue is located adjacently to helix F which partially embraces SRS-2 (see Fig. 4), this amino acid substitution could possibly disrupt the access channel and binding affinity of ligands and thus affecting the access and binding of 8-MOP and coumarin at the putative active site of CYP2A6.

Minimum effects on ligand binding observed in CYP2A6*16 indicate the lesser detrimental effect of R203S in this variant as compared to mutations in the other three alleles. This is also supported by our docking data that showed minimum change in active site volume. From the numerous molecular modeling and site-directed mutagenesis studies on CYP2A6 thus far, active site residues Q104, V113, F118, F205 (or F209), N290, I293, N297, T298, T305 and H477 have been reported to play critical role in the orientation and anchoring of coumarin for oxidation [14], [26]–[28] while neighbouring residue such as T212 is believed to be involved in directing the access of coumarin to the binding site [29] However, none of these contact sites involves residue R203 despite its location within the highly-conserved region of SRS2. In view of the data from our study and that of others, it is likely that R203S substitution have trivial effect on ligand binding for CYP2A6.

Kim and colleagues have previously shown that substitution of K476 with Ala, Arg or Asp in CYP2A6 had decreased binding affinity for coumarin (higher K_m_'s). Moreover, an additional mutant with K476E substitution had also exhibited low catalytic efficiency (∼8% that of the wild type k_cat_/K_m_ value) towards coumarin [30]. Such findings are consistent with our present observation that, variant CYP2A6*21 with its K476R substitution, possessed weaker affinity towards coumarin and 8-MOP as reflected by higher K_m_ and IC_50_ values (Table 1). The reduced catalytic efficiency of K476 mutants has been ascribed to perturbation of electron transfer as the residue is known to be involved in intermolecular electron transfer between CYP2A6 and reductase [30]. The K476E mutation, in particular, showed a greatly decreased rate of NADPH oxidation, suggesting that the low enzymatic activity may be caused by a decrease in utilization of electrons. Furthermore, this mutation is located close to Phe480, which is known to be an important residue forming part of the compact, hydrophobic active cavity of CYP2A6 as revealed by the X-ray crystallography study [14]. Thus it is likely that point mutation in CYP2A6*21, from K476 to another strongly basic substitute Arg, while not altering the local polarity, may have altered the interaction of F480 with the coumarin and 8-MOP in our study due to the subtle changes in the residue size. Our docking data further supported the important role of K476 as the mutation has caused the largest volume increase in CYP2A6 active site cavity and the loss of H bond, resulting in increased CDIE value.

Concurrent substitutions of amino acid residues adjacent to one another (D158E and L160I) in CYP2A6*22 yielded significantly reduced binding affinity (highest K_m_ value among the four variants, and 5.5-fold higher IC_50_ value) for coumarin and 8-MOP, implying a major compromise in its enzymatic activity. Both D158E and L160I substitutions are located in the D-helix, which appears to be exterior to the putative active site of CYP2A6 (Fig. 4). This, together with our docking data, has indicated that D158E and L160I residues were involved in the ‘long-range’ interactions resulting in enlarged active site volume which may affect the folding and conformational changes in the protein distant regions involved in ligand egress, binding, orientation as well as heme binding [9]. The role of L160 has also been supported by a previous study. Hadidi and co-workers reported that an individual homozygous for L160H mutation in CYP2A6 showed significantly enhanced coumarin 3-hydroxylation while lacking 7-hydroxylation activity [31]. This information again supports the postulation that structural elements outside of the active site may have an important role in controlling the protein catalytic activity.

In conclusion, we observed similar patterns of change in the IC_50_ and K_m_ values of CYP2A6*15, CYP2A6*16, CYP2A6*21 and CYP2A6*22 in the oxidation of coumarin. Except for CYP2A6*16, all variants showed considerably compromised binding affinity for coumarin and all of which also showed significantly higher IC_50_ values for 8-MOP in coumarin oxidation. In support of these *in vitro* data, our molecular docking study has also showed that the mutations have induced enlarged active site cavity in CYP2A6*15, CYP2A6*21 and CYP2A6*22 and loss of H bond between 8-MOP and active site residue Asn297 in all mutants. All these data indicate that structural changes induced by the mutations at different sequence locality have altered ligand binding within the CYP2A6 active site.

## Supporting Information

Table S1 **Changes in the active sites of CYP2A6 proteins due to mutations.**
(DOCX)
