# Supplementary material for: Inhibitory Potency of 8-Methoxypsoralen on Cytochrome P450 2A6 (CYP2A6) Allelic Variants CYP2A6*15, CYP2A6*16, CYP2A6*21 and CYP2A6*22: Differential Susceptibility Due to Different Sequence Locations of the Mutations
Source: PLoS One. 2014 Jan 27;9(1):e86230. doi: 10.1371/journal.pone.0086230 (PMC3903516; doi:10.1371/journal.pone.0086230)
Supplement: Table S1 — Changes in the active sites of CYP2A6 proteins due to mutations. (DOCX) [file pone.0086230.s001.docx]

**Table S1.** Changes in the active sites of CYP2A6 proteins due to mutations.

| Site | **CYP2A6*1** | | | | | **CYP2A6*15** | | | | | **CYP2A6*16** | | | | | **CYP2A6*21** | | | | | **CYP2A6*22** | | | | |
| --- | --- | --- | --- | --- | --- | --- | --- | --- | --- | --- | --- | --- | --- | --- | --- | --- | --- | --- | --- | --- | --- | --- | --- | --- | --- |
|  | X axis | Y axis | Z axis | Volume  (Å^3^) | Point count | X axis | Y axis | Z axis | Volume  (Å^3^) | Point count | X axis | Y axis | Z axis | Volume  (Å^3^) | Point count | X axis | Y axis | Z axis | Volume  (Å^3^) | Point count | X axis | Y axis | Z axis | Volume  (Å^3^) | Point count |
| 1 | 38.70 | 72.47 | 59.44 | 562.63 | 4501 | 36.58 | 72.49 | 62.54 | 394.13 | 3153 | 36.84 | 73.37 | 62.36 | 406.75 | 3254 | 38.12 | 73.03 | 62.68 | 351.13 | 2809 | 54.66 | 68.74 | 56.67 | 456.13 | 3649 |
| 2 | 51.20 | 80.22 | 28.93 | 240.88 | 1927 | 54.58 | 92.49 | 69.54 | 201.63 | 1613 | 51.84 | 88.62 | 67.86 | 343.75 | 2750 | 45.12 | 91.28 | 50.93 | 339.88 | 2719 | 35.91 | 73.99 | 63.42 | 398.38 | 3187 |
| 3 | 56.45 | 66.47 | 51.94 | 211.75 | 1694 | 42.58 | 89.24 | 54.79 | 200.75 | 1606 | 43.09 | 90.12 | 49.61 | 234.50 | 1876 | 57.12 | 65.28 | 52.68 | 187.50 | 1500 | 44.16 | 89.24 | 51.67 | 237.88 | 1903 |
| 4 | 54.45 | 92.47 | 66.94 | 163.13 | 1305 | 51.83 | 70.74 | 66.79 | 165.25 | 1322 | 72.09 | 96.62 | 57.61 | 173.50 | 1388 | 53.37 | 80.28 | 28.68 | 171.00 | 1368 | 54.66 | 82.24 | 29.67 | 215.25 | 1722 |
| 5 | 43.45 | 89.22 | 54.69 | 160.88 | 1287 | 54.33 | 78.74 | 29.29 | 164.13 | 1313 | 51.84 | 80.12 | 29.11 | 173.25 | 1386 | 51.87 | 90.03 | 70.93 | 162.38 | 1299 | 53.41 | 89.74 | 70.92 | 197.50 | 1580 |
| 6 | 43.45 | 89.22 | 54.69 | 160.88 | 1287 | 62.58 | 86.49 | 71.79 | 122.75 | 982 | 35.34 | 76.37 | 81.61 | 158.88 | 1271 | 62.12 | 85.78 | 72.18 | 153.38 | 1227 | 69.16 | 97.24 | 57.67 | 169.00 | 1352 |
| 7 | 35.95 | 77.47 | 81.19 | 125.38 | 100 | 58.08 | 64.49 | 52.04 | 110.25 | 882 | 57.09 | 68.87 | 53.11 | 132.38 | 1059 | 55.62 | 80.78 | 62.68 | 143.63 | 1149 | 55.16 | 79.74 | 61.42 | 135.13 | 1081 |
| **8** | **55.20** | **75.97** | **58.69** | **89.56** | **756** | **54.33** | **78.49** | **59.04** | **105.88** | **847** | **54.59** | **78.12** | **60.11** | **88.25** | **706** | **51.12** | **71.78** | **68.18** | **130.25** | **1042** | **36.41** | **76.49** | **82.67** | **120.75** | **966** |
| 9 | 59.70 | 76.22 | 72.44 | 107.25 | 858 | 67.83 | 96.24 | 55.54 | 103.50 | 828 | 55.59 | 93.37 | 53.86 | 73.38 | 587 | 36.12 | 77.53 | 82.68 | 122.88 | 983 | 63.91 | 90.49 | 71.67 | 116.50 | 932 |
| 10 | 39.70 | 92.47 | 42.69 | 88.00 | 704 | 54.33 | 92.24 | 55.29 | 84.75 | 678 | 65.59 | 92.37 | 71.86 | 70.00 | 560 | 68.62 | 96.53 | 56.43 | 98.50 | 788 | 54.91 | 94.99 | 50.42 | 112.13 | 897 |
| 11 | 57.45 | 91.47 | 52.44 | 77.25 | 618 | 49.33 | 85.74 | 67.29 | 61.13 | 489 | 44.59 | 63.62 | 40.36 | 67.00 | 536 | 63.37 | 72.03 | 39.93 | 74.88 | 599 | 33.41 | 66.24 | 70.42 | 92.63 | 741 |
| 12 | 33.20 | 64.97 | 73.19 | 74.88 | 599 | 36.83 | 75.24 | 83.29 | 60.38 | 483 | 35.34 | 78.87 | 38.61 | 65.00 | 520 | 79.12 | 93.03 | 60.43 | 72.63 | 581 | 69.66 | 72.74 | 51.67 | 75.00 | 600 |
| 13 | 44.20 | 62.97 | 44.19 | 61.88 | 495 | 34.58 | 77.99 | 38.54 | 56.00 | 448 | 59.59 | 81.87 | 70.11 | 63.13 | 505 | 44.87 | 64.03 | 43.68 | 59.38 | 475 | 78.41 | 93.24 | 60.42 | 57.13 | 457 |
| 14 | 49.45 | 75.72 | 65.69 | 59.13 | 473 | 71.33 | 76.74 | 48.29 | 53.25 | 426 | 33.84 | 66.62 | 69.86 | 55.25 | 442 | 33.62 | 77.28 | 38.43 | 53.88 | 431 | 38.41 | 91.24 | 75.67 | 56.50 | 452 |
| 15 | 76.20 | 83.47 | 51.19 | 55.38 | 443 | 78.08 | 84.49 | 51.04 | 51.88 | 415 | 76.84 | 87.37 | 50.36 | 39.00 | 312 | 55.87 | 91.03 | 56.68 | 52.00 | 416 | 62.41 | 68.74 | 39.42 | 47.63 | 381 |
| 16 | 30.95 | 82.72 | 49.19 | 51.13 | 409 | 43.58 | 63.74 | 42.54 | 51.13 | 409 | 70.09 | 69.87 | 54.86 | 38.63 | 309 | 31.62 | 86.03 | 49.68 | 44.50 | 356 | 35.41 | 77.24 | 38.92 | 46.50 | 372 |
| 17 | 41.20 | 68.72 | 32.94 | 37.00 | 296 | 77.58 | 96.99 | 60.54 | 48.63 | 389 | 31.84 | 86.12 | 50.36 | 36.38 | 291 | 37.12 | 90.78 | 75.18 | 44.00 | 352 | 58.41 | 79.49 | 71.42 | 45.38 | 363 |
| 18 | 33.20 | 77.97 | 38.44 | 36.88 | 295 | 50.83 | 64.49 | 77.04 | 46.50 | 372 | 59.59 | 67.87 | 68.61 | 35.00 | 280 | 70.62 | 75.53 | 48.43 | 36.13 | 289 | 30.91 | 85.49 | 49.92 | 40.75 | 326 |
| 19 | 45.45 | 86.72 | 44.19 | 34.50 | 276 | 40.58 | 91.24 | 42.04 | 40.63 | 325 | 64.09 | 69.62 | 39.11 | 32.38 | 259 | 41.87 | 73.78 | 36.43 | 27.38 | 219 | 41.41 | 70.24 | 33.92 | 34.38 | 275 |
| 20 | 70.45 | 78.97 | 47.94 | 30.75 | 246 | 32.08 | 67.49 | 71.79 | 31.00 | 248 | 70.09 | 78.62 | 49.11 | 31.13 | 249 | 65.87 | 87.03 | 65.68 | 27.25 | 218 | 44.16 | 64.24 | 42.92 | 34.25 | 274 |
| 21 | 78.95 | 96.47 | 64.19 | 26.75 | 214 | 52.83 | 76.24 | 39.04 | 26.38 | 211 | 53.84 | 77.37 | 39.86 | 29.38 | 235 | 70.12 | 71.28 | 53.43 | 26.88 | 215 | 76.91 | 85.49 | 49.92 | 28.25 | 226 |
| 22 | 66.70 | 94.47 | 71.19 | 26.25 | 210 | 48.08 | 76.99 | 78.79 | 26.25 | 210 | 47.84 | 74.87 | 67.11 | 26.63 | 213 | 51.62 | 67.78 | 77.68 | 26.13 | 209 | 48.91 | 67.24 | 78.92 | 26.25 | 210 |
| 23 | 44.20 | 78.47 | 37.94 | 22.88 | 183 | 57.08 | 71.24 | 49.29 | 25.50 | 204 | 41.59 | 71.37 | 34.11 | 25.75 | 206 | 57.62 | 83.78 | 49.18 | 23.63 | 189 | 53.91 | 77.99 | 40.42 | 22.00 | 176 |
| 24 | 49.95 | 61.22 | 75.19 | 22.75 | 182 | 42.08 | 71.49 | 34.04 | 24.38 | 195 | 66.09 | 70.12 | 66.86 | 20.13 | 161 | 36.12 | 64.53 | 68.43 | 20.88 | 167 | 58.66 | 88.49 | 56.42 | 20.25 | 162 |
| 25 | 54.70 | 95.72 | 40.19 | 22.75 | 182 | 41.58 | 83.49 | 75.54 | 23.13 | 185 | 65.09 | 72.62 | 43.61 | 16.13 | 129 | 35.62 | 71.53 | 46.18 | 20.25 | 162 | 35.91 | 92.99 | 37.92 | 17.63 | 141 |
| 26 | 52.45 | 86.97 | 76.94 | 22.63 | 181 | 66.33 | 70.24 | 66.29 | 22.13 | 177 | 43.84 | 77.12 | 79.86 | 15.88 | 127 | 51.37 | 60.03 | 75.43 | 18.38 | 147 | 50.91 | 59.74 | 74.67 | 16.38 | 131 |
| 27 | 44.95 | 78.97 | 54.19 | 20.75 | 166 | 43.58 | 78.99 | 39.54 | 18.00 | 144 | 51.84 | 66.37 | 78.11 | 15.25 | 122 | 51.87 | 72.28 | 54.68 | 16.13 | 129 | 58.41 | 83.99 | 48.17 | 16.38 | 131 |
| 28 | 57.95 | 71.47 | 49.19 | 17.50 | 140 | 55.08 | 96.24 | 41.54 | 14.38 | 115 | 42.34 | 60.62 | 70.61 | 14.38 | 115 | 30.87 | 68.53 | 72.43 | 13.13 | 105 | 33.66 | 86.49 | 36.92 | 14.13 | 113 |
| 29 | 32.95 | 85.72 | 37.69 | 15.63 | 125 | 27.58 | 84.24 | 85.54 | 14.00 | 112 | 62.59 | 80.62 | 53.86 | 13.75 | 110 |  |  |  |  |  |  |  |  |  |  |
| 30 | 40.70 | 85.72 | 61.94 | 14.38 | 115 | 70.58 | 71.49 | 54.54 | 13.63 | 109 | 39.84 | 86.62 | 61.11 | 12.63 | 101 |  |  |  |  |  |  |  |  |  |  |
| 31 | 78.70 | 88.97 | 61.19 | 12.50 | 100 | 33.08 | 86.49 | 51.79 | 13.38 | 107 |  |  |  |  |  |  |  |  |  |  |  |  |  |  |  |
| 32 |  |  |  |  |  | 63.83 | 69.99 | 42.29 | 13.38 | 107 |  |  |  |  |  |  |  |  |  |  |  |  |  |  |  |
